# Activation of the ankle musculature during the layup gesture in basketball players with chronic ankle instability, a case control study

**DOI:** 10.1371/journal.pone.0336380

**Published:** 2025-11-11

**Authors:** María Benito-de-Pedro, Raquel Delgado-Delgado, Ángela Aguilera-Rubio, Elena Sonsoles Rodríguez-López, Juan Nicolás Cuenca-Zaldívar, Maura Jiménez-Herranz, Ana Isabel Benito-de-Pedro

**Affiliations:** 1 Grupo de Investigación Fisioterapia y Salud (FYSA), Departamento de Fisioterapia, Facultad HM de Ciencias de la Salud, Universidad Camilo José Cela, Villanueva de la Cañada, Madrid, Spain; 2 Instituto de Investigación Sanitaria HM Hospitales, Madrid, Spain; 3 Department of Physical Therapy, Occupational Therapy, Rehabilitation and Physical Medicine, Faculty of Health Sciences, Rey Juan Carlos University, Alcorcón, Madrid, Spain; 4 Motion Analysis, Ergonomics, Biomechanics and Motor Control Laboratory (LAMBECOM), Department of Physical Therapy, Occupational Therapy, Rehabilitation and Physical Medicine, Faculty of Health Sciences, Rey Juan Carlos University, Madrid, Spain; 5 Universidad de Alcalá, Facultad de Medicina y Ciencias de la Salud, Departamento de Enfermería y Fisioterapia, Grupo de Investigación en Fisioterapia y Dolor, Alcalá de Henares, Spain; 6 Interdisciplinary Group on Musculoskeletal Disorders, Faculty of Sport Sciences, Universidad Europea de Madrid, Villaviciosa de Odón, Spain; 7 Research Group in Nursing and Health Care, Puerta de Hierro Health Research Institute - Segovia de Arana (IDIPHISA), Madrid, Spain; 8 Primary Health Center “El Abajón”, Las Rozas de Madrid, Spain; University of Tehran, IRAN, ISLAMIC REPUBLIC OF

## Abstract

Lateral ankle sprain (LAS) is a very common injury in the world of basketball. Its recurrence rate is very high, generating chronic ankle instability (CAI), accompanied by decreased muscle activation in the ankle musculature and lack of motor control. The aim is to assess the difference in the activation and the moment of greatest solicitation of the peroneus during the development of the layup basketball gesture and to assess the motor control at this moment of greatest solicitation, in patients suffering from CAI and in those who do not suffer from it. In this case-control study, a surface electromyography (sEMG) was conducted in the peroneus lateralis longus (PLL) and peroneus lateralis brevis (PLB) musculature during the layup gesture, on 58 female basketball players, 29 with and 29 without CAI. The presence of significant differences in the group: measurement interaction was found in the variables left PLL RMS left-hand layup and right PLL maximum peak left-hand layup (X^2^(6)=22.246, p = 0.001, X^2^(6)=12.99, p = 0.043) respectively, where higher normalized activation values are observed in CAI group. The post hoc test shows systematically higher percentage values in the CAI group (normalized results with MVC) and higher mean activity of peroneal muscle in patients without CAI. The greatest solicitation of activity in the layup gesture occurs in the impulse prior to the jump. The post hoc test shows systematically higher percentage values in the CAI group, results which non remain throughout the trials. The percentage of activation in the left PLL maximum peak right-hand layup is higher in the non CAI group with a large effect size in single measurements. In relation to motor control, a higher overall signal dispersion is observed in CAI group there is evidence of worse motor control.

## Introduction

Lateral ankle sprain (LAS) is the most common lower limb injury in physically active people [1], with women having approximately double the risk of suffering it than men [1,2]. More than 33% of LAS lead to chronic ankle instability (CAI) [3], a condition characterized by repetitive episodes of LAS; ongoing symptoms such as pain, muscle weakness, or reduced ankle range of motion (ROM) [4]. Neural factors have been identified [5,6], it´s proven evidence that individuals affected by CAI associated centrally mediated alterations resulting in sensorimotor deficits, static postural control, and gait dynamics [7,8]. These impairments in sensorimotor function have been shown to impair dynamic postural stability [9], movement mechanics [10] and neuromuscular control [11,12] during unilateral impulse and jump-landings [10]. This suggests that the presence of chronic ankle joint instability influences centrally mediated motor control strategies, resulting in maladaptive movement patterns [13].

Indoor pivot sports such as basketball, which involves frequent ankle-demanding actions as jumping and landing, impose significant loads on the ankle [14,15]. Given its specific physical demands, more than 70% of basketball players who suffer an ankle LAS will develop a high prevalence of CAI in basketball player [16].

Electromyography (EMG) studies conducted to date showing an altered muscle activation pattern in ankle muscle stabilizers in patients with CAI [17–20]. Peroneus lateralis longus (PLL) and peroneus lateralis brevis (PLB) are muscles whose main contribution is in the ankle movement and stability during demanding gestures like jumping in basketball [21,22]. Both are of great relevance in CAI patients due to their ability to counteract ankle inversion (eversion moment) through their activation [23].

We consider it highly relevant to understand the activation of the peroneal muscles during the most demanding moments of sports activity and the consequences of alterations in said activation in patients with CAI in relation to the development of new injuries. Although studies to date have shown that people with CAI have impaired neuromuscular control, especially when performing tasks involving anticipatory postural control and balance [19], no research has been found that demonstrates the extent to which peroneal muscle activation is altered during the most demanding moments of the basketball layup, one of the most common movements in this sport, and how this may affect the lead to new injuries in players with CAI.

Therefore, the aim of this study is to assess the difference in the activation of the PLL and PLB muscles during the development of the layup basketball gesture. As secondary objectives, it has been considered relevant to evaluate the moment of greatest solicitation of these muscles during layup gesture in control group and to assess the response at the same moment in CAI group and to assess the motor control in CAI group at the moment of greatest solicitation of the ankle and their response to a possible injury gesture.

## Methods

### Design

A case-control study was conducted. The entire procedure was carried out [24] for human experimentation purposes. The case control study followed the STROBE guidelines [25].

### Participants

In this study 58 female participants were selected (29 CAI group and 29 non CAI group) through non-probabilistic sampling in several amateur basketball teams in the Autonomous Community of Madrid.

For the sample size, with the data provided in Table 3 of the study by Tajdini et al. [26] accepting a risk α of 0.05 and a power greater than 80% without losses as it is a cross-sectional study, a sample of 58 is obtained with the PLL contact phase data for a calculated effect size of Cohen’s d = 0.828.

**Table 3:** Significant repeated measurements sEMG outcomes.

	T1	T2		T3	T4		T5	T6	T7
**CAI group**									
Right PLL maximum peak left-hand layup	76.481[31.192,100.915]	62.65[40.532,91.72]		75.376[31.184,104.499]	70.578[32.811,110.998]		77.317[28.428,105.957]	72.639[46.267,100.505]	69.158[39.332,138.016]
Right PLL RMS left-hand layup	26.386[11.758,34.281]	23.764[12.271,31.373]		29.44[12.583,36.205]	28.551[12.158,39.633]		26.897[11.975,36.741]	24.734[15.631,38.977]	27.324[14.714,40.251]
**Non-CAI group**									
Right PLL maximum peak left-hand layup	73.133[49.64,89.396]	68.085[49.031,117.21]		70.243[49.433,102.724]	53.372[33.268,76.302]		73.591[46.017,99.319]	64.324[45.427,95.505]	54.744[41.665,77.752]
Right PLL RMS left-hand layup	24.529[18.116,32.468]	26.615[21.048,39.32]		26.41[18.538,32.61]	23.122[12.667,27.803]		20.143[18.12,38.597]	20.158[16.518,31.78]	24.686[16.276,30.094]
**Omnibus test results**									
	**Group (** ^**a**^ **p value)**		**Measurements** (^**a**^**p value)**			**Group: measurements (** ^**a**^ **p value)**		$\eta_{p}^{2}$ **(95%CI)**	
Right PLL maximum peak left-hand layup	0.805		0.555			**0.043**		0.009 (0.004, 0.055)	
Right PLL RMS left-hand layup	0.68		0.43			**0.001**		0.007 (0.006, 0.054)	

Data expressed with mean±standard deviation; 95%CI: 95% confidence interval; all measurements in percentage of maximum voluntary contraction. T: number of trials. PLL: peroneus lateralis longis. RMS: root mean square.

^a^significant if p < 0.05 (shown in bold).

All patients were informed before the study by means of a patient information sheet, after which they signed an informed consent form. This study was prospectively registered at ClinicalTrials.gov with the number NCT04157426 and evaluated and approved by the Ethics Committee 15_24_CQSI, of the Camilo José Cela University (Madrid). The study followed the Ethics Criteria of the Declaration of Helsinki [24].

Basketball players with (CAI group) and without (non CAI group) CAI were recruited. The recruitment period for participants was from November 2023 to June 2024. For both groups all participants had to meet the following inclusion criteria: volunteers aged 18–45 years, physical activity level of at least 3 hours per week and for the CAI group all participants should achieve the criteria recommended by the International Ankle Consortium [27]:1) first sprain more than one year ago (medical diagnosis), 2) no sprain in the 6 weeks prior to the test, 3) decreased self-reported function (Foot and Ankle Ability Measure (FAAM) Sport Score < 80%, 4) scored ≥ 11 on the Functional Ankle Instability Identification (IdFAI) questionnaire [27].

Exclusion criteria: having undergone lower limb surgery and/or diseases that may influence neuromuscular control [28], as well as suffering from any type of incapacity to complete the proposed sporting gesture. Patients of non CAI group should not have a history of ankle sprains.

A total of 58 female basketball players participated in this study, without any loss during the measurements.

### Data collection and processing

Muscle activity was recorded using an mDurance R surface EMG (sEMG) system (mDurance Solutions SL, Granada, Spain), a portable sEMG system that consists of three parts [29]: (i) A Shimmer3 EMG unit (Realtime Technologies Ltd., Dublin, Ireland), which is a bipolar sEMG sensor for muscle activity acquisition; (ii) the mDurance mobile application (Android), which receives data from the Shimmer unit and sends it to a cloud service [29]; (iii) the mDurance cloud service, where the sEMG signals were stored, for subsequent evaluation and digitally filtered using a fourth-order “Butterworth” band pass filter between 20 and 450 Hz. For reduced movement artifacts, a high-pass cutoff frequency of 20 Hz [30].

Previously, the skin must be prepared, Surface EMG for Non-Invasive Muscle Assessment (SENIAM) recommends shaving the area of skin where the electrodes are to be placed, if necessary. Afterwards, clean the shaved area with alcohol and allow it to dry before placing the electrodes.

**Fig 1:**
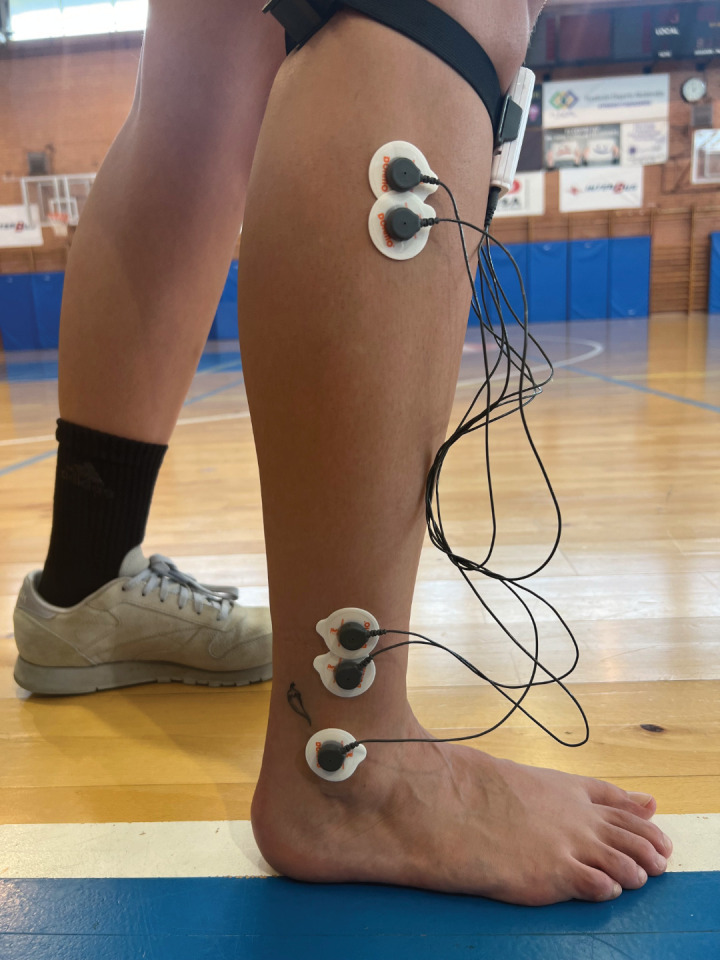
Placement of surface electrodes according to SENIAM protocol.

Surface electrode placement was performed following the recommendations of SENIAM [31] bilaterally and parallel to the direction of the muscle fibres (Fig 1). For the PLL, electrodes need to be placed at 25% on the line between the tip of the head of the fibula to the tip of the lateral malleolus and for the PLB electrodes need to be placed at 25% of the line from the tip of the lateral malleolus to the fibula-head with an electrode distance in both of 2 cm. The reference electrode needs to be placed on/around the ankle [31].

### Protocol of measurement

Firstly, anthropometric variables and personal information, training hours of the participants as well as ankle and lower limb injuries were collected through a Microsoft Forms.

In this study, changes in the sEMG signal of PLL and PLB in patients of CAI and control group were investigated in the performance of basketball layup gesture. These changes in signal amplitude (root mean square (RMS)) reflect the level of activation during the layup gesture in the PLL and PLB muscles.

**Maximum voluntary contraction**: in the first part of the study, participants were asked to perform an isometric maximum voluntary contraction (MVC) [32], both PLL and PLB towards eversion and in the supine position, from the inversion position. Three maximal attempts of 5 seconds each, separated by 1 minute of rest, were performed. The best performance was chosen for statistical analysis [33].

Participants were verbally encouraged as we conducted the assessment. The purpose of this test was to compare maximal amplitudes with submaximal amplitudes [34].

**Development of layup gesture**: in the second part, the participants performed the sport gesture of layup, always with the same ball, with a perimeter size of 72.4–73.7 cm as per National Basketball Association standards [35], and the basket is always at the same high, 305 cm.

**Fig 2:**
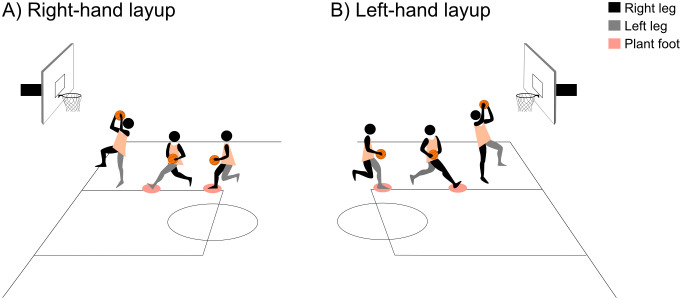
A) Right-hand layup gesture. B) Left-hand layup gesture.

The participants first warmed up for 10 minutes on the court. This was followed by 1 series of 7 right-hand layups, (first step starting with the right leg and second starting with the left leg) (Fig 2A) and another series of 7 left-hand layups (first step starting with the left leg and second starting with the right leg) (Fig 2B), regardless of the dominant side, which was determined using a ball kick test [36]. In between, the players were asked to rest for 5 minutes [37].

The drive to the basket is a sporting gesture that consists of taking 3 steps towards the basket, (i) a short step towards the basket, (ii) a long step towards the basket, and (iii) a final jump (Fig 2).

### Statistical analysis

The statistical analysis was performed with R Ver. 4.1.3. (R Foundation for Statistical Computing, Institute for Statistics and Mathematics, Welthandelsplatz 1, 1020 Vienna, Austria).

The level of significance was set at p < 0.05. Qualitative variables were described in absolute values and frequencies and quantitative variables with mean and standard deviation or median [interquartile range] in the outcome variables. In each group, the Shapiro-Wilk test was applied to assess the distribution of variables.

For baseline clinical-demographic variables as well as sEMG parameters, the presence of significant differences between the two groups was analysed using the Mann-Withney U test for non-parametric or a Student’s t-test for parametric variables (after checking the assumption of homogeneity of variances using Levene’s test) according to their distribution, and with Fisher’s exact test in the case of qualitative variables. The effect size was calculated with the non-parametric r statistic, defined as small (<0.4), moderate (0.4–0.6) and large (>0.6).

The repeated measurements sEMG variables with the tests were analysed using a robust repeated measures model with two factors, between (groups) and within (measurements) by calculating a robust Wald-type pseudo-statistic [38]. For post hoc tests, the Mann-Whitney U test with Bonferroni correction was applied. The effect size was calculated with the $\eta_{\text{p}}^{\text{2}}$ statistic obtained by bootstrap (bootstraped partial eta squared), defined as small (<0.06), moderate (0.06–0.14) and large (>0.14).

Finally, a cluster analysis [39] was performed on the complete sEMG record with the objective of characterizing the muscle synergies in both groups.

## Results

A total of 58 female players participated in the study with a mean age of 29.19 ± 6.98 years and a BMI of 21.87 ± 1.64 with no differences between the two groups (Table 1).

**Table 1:** Clinical and demographic characteristics of the participants.

		Overall	Non-CAI group	CAI group	Average difference (95% CI)	Levene´s test (^a^p value)	^a^p value
n		58	16	42			NA
**Clinical and demographic characteristics**							
Age (years)		29.19 ± 6.98	26.69 ± 3.52	30.14 ± 7.74	3.455 (0.484, 6.427)		0.191
Weight (kg)		66.46 ± 7.23	68.69 ± 6.42	65.61 ± 7.41	−3.08 (−7.097, 0.936)	0.324	0.149
Height (m)		1.74 ± 0.07	1.75 ± 0.07	1.74 ± 0.08	−0.015 (−0.057, 0.027)	0.597	0.498
BMI (kg/m^2^)		21.87 ± 1.64	22.31 ± 1.15	21.70 ± 1.77	−0.612 (−1.411, 0.187)	0.239	0.206
Foot size (ES)		41.32 ± 2.65	42.06 ± 2.66	41.04 ± 2.62	−1.027 (−2.626, 0.572)		0.196
Dominant arm, n (%)	Left	2 (3.4)	1 (6.2)	1 (2.4)			0.479
	Right	56 (96.6)	15 (93.8)	41 (97.6)			NA
Dominant leg, n (%)	Left	4 (6.9)	2 (12.5)	2 (4.8)			0.303
	Right	54 (93.1)	14 (87.5)	40 (95.2)			NA
Forward foot free kick, n (%)	Left	2 (3.4)	1 (6.2)	1 (2.4)			0.712
	None	10 (17.2)	3 (18.8)	7 (16.7)			NA
	Right	46 (79.3)	12 (75.0)	34 (81.0)			NA
**Clinical characteristics of CAI group**							
Sprain number last 6 months				0.40 ± 0.59			
Last sprain side, n (%)	Left			20 (47.6)			
	Right			22 (52.4)			NA
Months since last sprain				27.96 ± 54.27			
Sprain level, n (%)	I			9 (21.4)			
	II			10 (23.8)			NA
	III			8 (19.0)			NA
	Unknown			15 (35.7)			NA
Received sprain physical therapy treatment, n (%)	No			5 (11.9)			
	Yes			36 (85.7)			NA

NA, not applicable; BMI, body mass index; ES, European standard.

Data expressed with mean±standard deviation or with relative absolute values (%); 95% CI: 95% confidence interval.

^a^significant if p < 0.05.

Table 2 shows the data expressed in µV of maximum voluntary activation of both peroneal muscles, showing greater activation by the non-CAI group.

**Table 2:** Maximum voluntary contraction assessment.

	Non-CAI group	CAI group
Right PLL MVC	863.14 [553.23, 1297.76]	703.37 [392.60, 1135.09]
Left PLL MVC	717.50 [415.03, 1606.09]	533.19 [374.42, 1051.99]
Right PLB MVC	639.09 [551.62, 1308.71]	639.32 [423.41, 841.89]
Left PLB MVC	765.51 [605.44, 1123.51]	592.50 [472.06, 889.73]

PLL: peroneus lateralis longus; PLB: peroneus lateralis brevis; MVC: Maximum voluntary contraction Data expressed with median [interquartile range].

### sEMG findings

In Table 3, where each layup is named from first (T1) to last (T7), significant differences in the group: measurements interaction were found in the variables right PLL RMS left-hand layup and right PLL maximum peak left-hand layup (X^2^(6)=22.246, p = 0.001, X^2^(6)=12.99, p = 0.043) respectively, (significant differences are shown in bold) where higher activation values are observed in CAI group.

**Fig 3:**
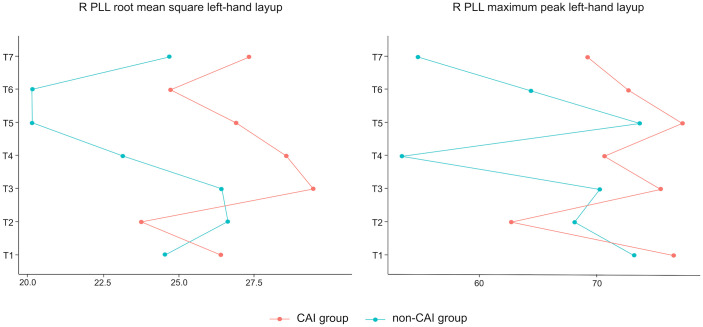
Most significant muscle activation values. All measurements in percentage of MVC. R PLL: right peroneus lateralis longus; T: number of trials.

The most significant muscle activation values are shown in Fig 3. The post hoc tests don’t detect significant differences between groups throughout the repeated measurements due to the high number of repeated measurements that correct the level of significance. Although it does not reveal significant differences, the interaction plot shows systematically higher percentage values during the sporting gesture in the CAI compared with the non -CAI group. This indicates that in CAI group, muscle percentage activation is higher because the MVC is lower.

### Muscle synergies analysis

**Fig 4:**
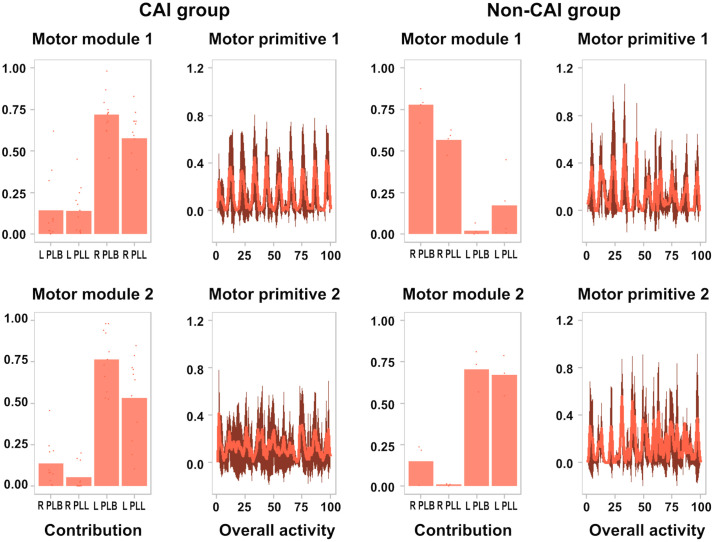
Layup muscle synergies. L PLB, left peroneus lateralis brevis; L PLL, left peroneus lateralis longus; R PLB, right peroneus lateralis brevis; R PLL, right peroneus lateralis longus.

During the layup two muscular synergies appear depending on which is the leg of impulse, that is, the opposite leg to which the layup gesture begins, with a strong activation of the peroneal muscles of the same (75% approximately), while the opposite leg remains practically inactive (less than 25%), with a particularity, the basal activation of that leg that remains in the air is greater in the CAI group than in the non-CAI (near to 0% in the last), probably due to the deficit they present in the passive structures, which forces them to maintain a basal activation of security (Fig 4).

In all the synergies evaluated (Fig 4) it is observed that the dispersion of the sEMG signal is greater in the CAI group. This suggests that it is more difficult for them to create a high level of depolarization, which indicates a worse motor control in the sport gesture.

### Single measurements sEMG findings

The percentage of activation in the left PLL maximum peak right-hand layup is higher in the non CAI group with a large effect size (Table 4).

**Table 4:** Significant single measurement sEMG outcomes.

	Non-CAI group	CAI group	Average difference (95% CI)	^a^p value	r (95% CI)
Left PLL maximum peak right -hand layup	110.58 [104.61,128.65]	99.27 [75.98,113.06]	−21.082 (−35.202, −6.961)	**0.013**	0.751 (0.146, 1.356)

Data expressed with median [interquartile range]; 95%CI: 95% confidence interval; Ratio expressed in µV; PLL: peroneus lateralis longus.

^a^significant if p < 0.05 (shown in bold).

The average peak activation moments throughout the layups remain higher in non CAI group, most likely due to the fatigue suffered by the peroneal muscles of patients with CAI, as described previously, which prevents them from maintaining high activation peaks throughout the layups, something that does occur in patients non CAI.

## Discussion

With the results described, the research questions that were initially proposed have been answered. These results offer us a novel and clear vision, which until now had not been investigated, about how the presence of CAI in basketball players affects the performance of layup basketball gesture. It is also clear that having CAI, in a sport like basketball, generates a great predisposition to suffer new injuries.

The results of our research show that, after starting from a homogeneous group, the measurement of the activity of the PLB and PLL muscles during layup gesture, shows much higher maximum activation values in the non CAI group (single measurement), likewise happens when measuring the MVC. Similar results were shown by Wang [40] and Thompson et al. [41]. This situation suggests that, at times of greater muscular solicitation, CAI group respond less well to generate contraction and to generate active stability in this movement, a situation that may lead to a recurrence in the event of an injurious gesture.

Our normalized results, with lower MVC in the CAI group than in the non CAI group, show a higher percentage of peak activation in peroneal muscles, when the impulse is requested, in CAI group. This percentage drops below the non CAI group when we calculate the average of peaks of all layups, and this is probably due to fatigue, results that coincide with those obtained by Wang et al. in their study in 2023 [40]. In this sense, Méndez-Rebolledo et al. shown in their recent study of 2024 [17] that the posterior compartment of the PLL (assessed with high-resolution sEMG) is inhibited in patients with CAI.

It is possible to think that this situation may be related to the presence in these patients with CAI of Myofascial Trigger Points (MTrP) in the peroneal of the affected ankle. Repetitive ankle injuries are proposed as one potential mechanism for activation MTrP [42] and this MTrP have been related to the presence of altered motor control patterns [43] and accelerated muscle fatigability [44,45] in the affected and related musculature.

In this regard, it is worth highlighting the conclusions obtained in the recent study by Watanabe et al. in 2024, which shown and increased activation of the intrinsic musculature of the foot, specifically of the abductor hallucis in patients with CAI, probably in an attempt to increase stabilisation by compensating for deficits during extrinsic ankle muscle activation [46]. Similar results were obtained by McLeod and Gribble [47], and Ahn et al. in 2020 [48] in flexor digitorum longus and tibialis anterior and by the authors Serra et al. in medial gastrocnemius and tibialis anterior muscles [49].

Our results also reveal that non CAI group in unloading moments are maintained during layup with lower maximum activation peaks and with fewer oscillations (lower resting values), than CAI group, which in the absence of functional passive stabilisers, could be interpreted as a defence mechanism, results similar to those shown in the publications of Cho et al. [50], Koldenhoven et al. [51] and Fraser et al. [52].

For all synergies assessed during the layup impulse, sEMG signal dispersion is observed to be higher in the CAI group, in the impulse moment, indicating worse motor control, results that are in line with those obtained by Simpson et al. [11]. In contrast, Kim et al. in their recent research from 2023, show similar motor control of the ankle musculature (including PLL) in CAI group and non CAI group, in the take-off/impulse (analysed by us) and landing phases [53].

Responding in this way to the objective of the research, the execution of layup involved stability, muscle control coordination in the ankle, and we must also take account the speed of the gesture and the final impulse, resulting in a handicap for patients of CAI group as they are less able to accurately execute actions that require precise footwork and jumps [54], leading to worse sports performance and greater predisposition to new ankle injuries.

As a novelty aspect of our study, the results show in CAI group lower maximum activation values (MVC), so despite the CAI group having higher peaks of normalized activity, throughout the layup gesture, a commonly performed basketball movement repeated frequently during a match or training, this muscle activity, at times of greatest demand such as the impulse or take off, decreases. This can lead to decreased effectiveness of the movement, as well as sprain recurrences and new injuries, as these patients have stability problems, which are also not adequately compensated by the peroneus. This, coupled with the dispersion of the EMG signal in CAI group, indicative of poorer motor control and, in turn, a greater predisposition to new injuries, makes specific peroneal muscle treatment and training necessary in all patients diagnosed with CAI, both to improve the effectiveness of the sports movement and to prevent relapses or new injuries.

### Strengths and limitations

We consider one of the main strengths of this study to be the large number of players with CAI, which improves the quality of our results.

This article has limitations that should be pointed out. Firstly, during the measurements could have influenced the outcome of the measurements.

Sometimes the measurements were taken prior to training, sometimes during and even at the end of training, a situation that can also condition the player’s muscle fatigue.

We want to highlight too, the fact that almost all of our players were right-handed and that they perform the layup gesture almost always on the same side in their sports routine.

Finally, in this study, an evaluation of the MTrP present in the PLL and PLB muscles was not performed, so we could not compare our results with those of other studies such as that of Simons D.G et Ge H-Y. et., al, which highlights the changes in muscle activation in the presence of these MTrP.

## Conclusions

In relation to the assessment of the average activation percentage of PLL and PLB during all layup gesture, a higher average of both is observed in patients of non CAI group.

The normalized results show a higher peak activation percentage in CAI group, because their MVC is lower than the MVC of non CAI group.

The greatest solicitation of activity with normalized results, in the layup gesture occurs in the impulse prior to the jump in CAI group, but when this repetition is maintained over time (match or training), the non CAI group maintains a higher percentage of activation, which implies in CAI group a lower efficacy for the development of the layup gesture along the match or training.

In moments of rest (foot in the air during the impulse of layup gesture), both peroneal muscles maintain a higher basal activity in CAI group, which could be interpreted as a defence mechanism in the absence of functional passive stabilisers.

In relation to motor control, there is evidence of EMG signal dispersion in layup gesture and even in the MVC test, much greater in CAI group, which shows a less homogeneous maintenance of the activity during the solicitation of the muscle, interpreted as worse motor control in CAI group, which makes them predisposed to repetitive injuries in that ankle.
